# HEPATOCELLULAR CARCINOMA: DIAGNOSIS AND OPERATIVE MANAGEMENT

**DOI:** 10.1590/0102-6720201700040011

**Published:** 2017

**Authors:** Marcio F. CHEDID, Cleber R. P. KRUEL, Marcelo A. PINTO, Tomaz J. M. GREZZANA-FILHO, Ian LEIPNITZ, Cleber D. P. KRUEL, Leandro A. SCAFFARO, Aljamir D. CHEDID

**Affiliations:** 1 Postgraduate Program in Surgical Sciences;; 2 Unit of Hepatobiliary Surgery and Liver and Pancreas Transplantation, Division of Gastrointestinal Surgery;; 3 Interventional Radiology Unit, Hospital de Clínicas de Porto Alegre, Federal University of Rio Grande do Sul, Porto Alegre, RS, Brazil

**Keywords:** Hepatocellular carcinoma, Diagnosis, Liver resection, Liver transplantation, Carcinoma hepatocelular, Diagnóstico, Ressecção hepatica, Transplante hepático

## Abstract

***Introduction*:**

Hepatocellular carcinoma is an aggressive malignant tumor with high lethality.

***Aim*:**

To review diagnosis and management of hepatocellular carcinoma.

***Methods*:**

Literature review using web databases Medline/PubMed.

***Results*:**

Hepatocellular carcinoma is a common complication of hepatic cirrhosis. Chronic viral hepatitis B and C also constitute as risk factors for its development. In patients with cirrhosis, hepatocelular carcinoma usually rises upon malignant transformation of a dysplastic regenerative nodule. Differential diagnosis with other liver tumors is obtained through computed tomography scan with intravenous contrast. Magnetic resonance may be helpful in some instances. The only potentially curative treatment for hepatocellular carcinoma is tumor resection, which may be performed through partial liver resection or liver transplantation. Only 15% of all hepatocellular carcinomas are amenable to operative treatment. Patients with Child C liver cirrhosis are not amenable to partial liver resections. The only curative treatment for hepatocellular carcinomas in patients with Child C cirrhosis is liver transplantation. In most countries, only patients with hepatocellular carcinoma under Milan Criteria are considered candidates to a liver transplant.

***Conclusion*:**

Hepatocellular carcinoma is potentially curable if discovered in its initial stages. Medical staff should be familiar with strategies for early diagnosis and treatment of hepatocellular carcinoma as a way to decrease mortality associated with this malignant neoplasm.

## RESUMO

*****Introdução***:**

O carcinoma hepatocelular é neoplasia maligna agressiva com elevada morbidade e mortalidade.

***Objetivo*:**

Revisão sobre a fisiopatologia, o diagnóstico e o manejo do carcinoma hepatocelular nos vários estágios da doença.

***Método*:**

Revisão da literatura utilizando a base Medline/PubMed e literatura adicional.

***Resultados*:**

O carcinoma hepatocelular é geralmente complicação da cirrose hepática. As hepatites virais crônicas B e C também são fatores de risco para o surgimento do carcinoma hepatocelular. Quando associado à cirrose hepática, ele geralmente surge a partir da evolução de um nódulo regenerativo hepatocitário que sofre degeneração maligna. O diagnóstico é efetuado através de tomografia computadorizada de abdome com contraste endovenoso, e a ressonância magnética pode auxiliar nos casos que não possam ser definidos pela tomografia. O único tratamento potencialmente curativo para o carcinoma hepatocelular é a ressecção do tumor, seja ela realizada através de hepatectomia parcial ou de transplante. Infelizmente, apenas cerca de 15% dos carcinomas hepatocelulares são passíveis de tratamento cirúrgico. Pacientes portadores de cirrose hepática estágio Child B e C não devem ser submetidos à ressecção hepática parcial. Para esses pacientes, as opções terapêuticas curativas restringem-se ao transplante de fígado, desde que selecionáveis para esse procedimento, o que na maioria dos países dá-se através dos Critérios de Milão.

***Conclusão*:**

Quando diagnosticado em seus estágios iniciais, o carcinoma hepatocelular é potencialmente curável. O melhor conhecimento das estratégias de diagnóstico e tratamento propiciam sua identificação precoce e a indicação de tratamento apropriado.

## INTRODUCTION

Hepatocellular carcinoma (HCC), hepatocarcinoma or hepatoma accounts for more than 90% of all cases of primary liver cancer^20^. It is the sixth most common type of cancer worldwide and has shown a significant increase in its incidence, becoming third leading cause of cancer-related mortality^27^.

This article aims to review the pathophysiology, diagnosis and management of hepatocellular carcinoma in the various stages of the disease.

## METHODS

A bibliographic survey was carried out in the following indexed databases: Latin American Literature in Health Sciences (Lilacs), Scientific Electronic Library Online (SciELO) and Pubmed. For the survey of the articles, the controlled descriptors of the Virtual Health Library were used through Decs (Descriptors in Health Sciences) consisting of “hepatocellular carcinoma” AND “diagnosis” AND AND “hepatic resection” AND “hepatic transplantation”, and Mesh (Medical Subject Headings) consisting of “hepatocellular carcinoma” AND “diagnosis” AND “liver resection” AND “liver transplantation”

## RESULTS

### Etiology 

Cirrhosis, defined as fibrosis associated with nodular regeneration is considered a premalignant condition^20^. In Western countries, including Brazil, 70-80% of HCC cases are associated to cirrhosis secondary to chronic infection with either hepatitis B or C viruses^5^. Alcohol also is an important predisposing factor to cirrhosis and HCC. In virtually all cases of HCC associated with the presence of HBV, there is integration of the HBV genome into the hepatocyte DNA^20^. In addition to that there are patients with negative serology for B-virus and presence of HBV in the tumor^18^. Non-alcoholic steatohepatitis is a risk factor for liver cirrhosis and HCC especially in obese patients. Other risk factors for the onset are aflatoxins and metabolic diseases, such as hemochromatosis, type I glycogenosis, alpha-1-antitrypsin deficiency, Wilson’s disease and porphyrias. HCC can rarely occur without recognized risk factors^18^. Fibrolamellar type, for example, is most often unrelated to previous cirrhosis or viral liver disease^18^.

### Pathology 

HCC may present as a unifocal, multifocal, or diffusely infiltrative tumor^20^. All patterns demonstrate broad potential for vascular invasion. When associated with cirrhosis, HCC usually arises from malignant transformation of a regenerative nodule. There is stimulation to angiogenesis, and the tumor receives abundant arterial vascularization. The mean tumor duplication time is about 200 days^12^. This time decreases as tumor increases. With up to 3 cm in size, HCC is generally well differentiated, encapsulated, and has low potential for blood vessel invasion. When it reaches approximately 5 cm in size, the nodule begins to loose differentiation and to exhibit microscopic vascular invasion^3, 16^ acquiring capacity to generate metastases.

### Natural history, screening and diagnosis 

At the time of diagnosis, only a minority of HCC cases are amenable to potentially curative intervention. When operative intervention is not possible, the tumor usually grows as a tumor that reduces liver function and generates intra- and extrahepatic metastases (mainly to the lungs and bones)^20^. In these cases, death usually occurs in a mean time of 10 months, being caused by tumor cachexia, hemorrhage of esophageal or gastric varices, hepatic insufficiency or, more rarely, hemoperitoneum secondary to tumor rupture^20^.

Patients with risk factors for HCC should undergo periodic HCC screening. The cost-effectiveness of screening has been widely demonstrated^3^. In addition, some studies suggest that HCC screening would confer increased survival for patients with hepatic cirrhosis^3^.

Utilization of ultrasonography for screening does not involve the use of ionizing radiation and is widely available. Its sensitivity varies from 60-80%, with a specificity greater than 90% in patients with cirrhosis^8^. Therefore, it is the method of choice for HCC screening in patients with hepatic cirrhosis, and should be performed every six months. Some defend that it should be combined with dosing of serum alpha-fetoprotein.

The definitive diagnosis of HCC is achieved through contrast enhanced CT scan of the abdomen and/or MRI. Contrast enhanced CT and MRI scans usually reveal a nodule with important enhancement in the arterial phase, becoming hypersensitive or hyperattenuating (wash in). In the portal and late phases, HCC usually undergoes rapid elimination of the contrast (wash out), becoming hypodense or hypoattenuating in comparison to the rest of the liver parenchyma.

Sensitivity of CT scan is 68% and its specificity is 93%^11^. MRI presents similar results, with a sensitivity of 81% and a specificity of 85%^11^. Accuracy of MRI can be significantly increased when new hepatospecific sequences and contrast media are utilized.

The American Association for the Study of Liver Diseases developed the following recommendations for patients with hepatitis B or cirrhosis and a liver nodule: a) nodules smaller than 1 cm identified by ultrasonography should be followed at three month intervals, and if there is no evidence of growth in two years, the nodule should be considered as a regenerative nodule; b) nodules greater than 1 cm should be evaluated by contrast enhanced dynamic studies - either CT or MRI - in order to identify typical characteristics of HCC, such as marked impregnation in the arterial phases with venous contrast wash out; b1) if typical malignant features are identified, there is no need for additional methods and the diagnosis of HCC is established; b2) if there are no typical features at the dynamic study, a second dynamic or even biopsy study may be considered.

Percutaneous HCC biopsy should be avoided as there may be tumor spread in the percutaneous needle path (this risk is about 3%)^8^. In addition, there is risk of hemoperitoneum secondary to puncture. When HCC is diagnosed, chest CT is recommended for staging^8^. Occurrence of extrahepatic metastases contraindicates liver resection and transplantation (LT)^18^.

### Staging of hepatocellular carcinoma

Two HCC staging systems can be utilized: Barcelona Clinic Liver Cancer (BCLC) staging system (Table 1A) and the American Joint Committee on Cancer (AJCC) staging system (Table 1B)^24^. 

**TABLE 1:** Staging: A) Barcelona Clinic Liver Cancer (BCLC) System; B) TNM

A	
B	**T - Primary Tumor** Tx Primary tumor cannot be assessed T0 No evidence of primary tumor T1 Solitary tumor without vascular invasion T2 Solitary tumor with vascular invasion or multiple tumors, none > 5 cm T3a Multiple tumors > 5 cm T3b Single tumor or multiple tumors of any size involving a major branch of the portal or hepatic vein T4 Tumor(s) with direct invasion of adjacent organs other than gallbladder or with visceral peritoneum **N - Regional lymph nodes** NX Regional lymph nodes cannot be assessed N0 No regional lymph node metastasis N1 Regional lymph node metastasis **M - Distant metastasis** M0 No distant metastasis M1 Distant metastasis **Anatomic stage/prognostic groups**

### Treatment

The most effective treatments for HCC are liver resection and LT. Ablative therapies [radiofrequency ablation (RFA), microwave ablation, and percutaneous ethanol injection] have a small potential for cure restricted to HCC smaller than 2 cm in size (BCLC 0 stage)^3^. However, ablative therapies are most commonly employed in patients to whom both liver resection and transplantation are contraindicated due to prohibitive surgical risk (advanced age and presence of clinical comorbidities).

Indications for surgical resection or LT in HCC should take into account factors such as number of tumors, tumor size, presence of cirrhosis, and also the experience of the surgeon with liver resections in patients with cirrhosis. Availability of organ donors is also a factor to be considered in deciding between liver resection or listing for transplantation.

Due to impairment of function and regeneration capacity, cirrhotic liver reacts poorly to partial resections^10^. In patients with cirrhosis, a thorough evaluation of liver function is required. In order to correctly stratify these patients, the degree of portal hypertension should be estimated. Child-Pugh classification (Table 2) and evaluation of serum albumin, serum bilirubin, INR, ascites and encephalopathy also are performed.

**TABLE 2:** Child-Pugh classification

Criteria	1 point	2 points	3 points
Bilirubin (mg/dl)	<2	2-3	>3
Albumin (g/dl)	>3,5	2,8-3,5	<2,8
Prothrombin time *(s) /* INR	1-3 / <1,7	4-6 / 1,7-2,3	>6 / >2,3
Ascites	None	Controlled	Refractory
Encephalopathy	None	Mild to moderate (grade 1 or 2) or suppressed with medication	Severe (grade 3 or 4) or refractory

Child A: Liver cirrhosis and total score 1 - 6

Child B: Liver cirrhosis and total score 7 - 9

Child C: Liver cirrhosis and total score 10 - 15

CT volumetry of the liver is critical and should be performed in virtually all patients who are potential candidates for partial liver resections. CT volumetry of the liver would not be necessary in patients presenting with a small HCC located in the anterior segments of the liver who are deemed as candidates to wedge liver resection or subsegmentectomy. Indocyanine green clearance test can be used whenever available. Cirrhotic patients whose indocyanin green clearance is up to 14% within 15 min after infusion may undergo partial liver resections^10^.

Therapeutic possibilities (resection, listing for LT, or non-surgical treatment for HCC) should be discussed with the patient and its relatives. Patient’s willingness must always be respected.

Patients with HCC and without cirrhosis are generally candidates for partial hepatic resections. On the other hand, presence of esophageal varices or other signs of portal hypertension such as ascites and significant thrombocytopenia (<100,000/mm^3^) generally contraindicate partial liver resection. Therefore, patients with Child B stage cirrhosis rarely are candidates to partial liver resections. Patients with Child C cirrhosis and HCC are potential candidates for LT, whenever selectable through the Milan Criteria (single lesion up to 5 cm or up to three lesions all less than 3 cm).

The extent of hepatic and vascular involvement by the tumor should be assessed should be carefully assessed by liver imaging. Detection of vascular invasion and/or tumor thrombus in branches of the inferior vena cava, hepatic veins or portal vein is performed through abdominal CT (preferably with concomitant CT angiography) and/or magnetic resonance imaging. Macrovascular invasion of branches of the portal vein or hepatic veins is a contraindication to LT^8^.

Preoperative clinical and laboratory evaluation for major operation is required. In addition, in order to undergo partial hepatic resection, the patient must have a Performance Status of zero or 1 on the Eastern Cooperative Oncology Group scale (Table 3).

**TABLE 3:** Performance Status Scale (Eastern Cooperative Oncology Group)

0	Fully active, able to carry on all pre-disease performance without restriction
1	Restricted in physically strenuous activity but ambulatory and able to carry out work of a light or sedentary nature
2	Ambulatory and capable of all selfcare but unable to carry out any work activities; up and about more than 50% of waking hours
3	Capable of only limited selfcare; confined to bed or chair more than 50% of waking hours
4	Completely disabled; cannot carry on any selfcare; totally confined to bed or chair

### Partial liver resection

After assessment of tumor extent and clinical conditions, an evaluation of hepatic functional reserve should be performed. Patient is classified according to Child-Pugh score. In addition, CT volumetry of the liver is warranted. The degree of portal hypertension should be assessed, ideally by measuring the hepatic venous pressure gradient, which should not exceed 10 mmHg.

Indirect methods of measuring portal pressure comprise evaluation of the presence of esophageal varices by endoscopy or even of varices in other sites of the portal system by CT of the abdomen. The serum platelet count is another method for indirect assessment of the degree of portal hypertension. When serum platelet count is decreased in patients with cirrhosis, especially when platelet count is lower than 100,000/mm^3^, it usually indicates portal hypertension. The presence of portal hypertension, detected by any of the above methods, constitutes a relative contraindication to the performance of partial hepatectomy^10^.

A study evaluated the results of partial hepatic resection for the treatment of HCC in 543 patients operated at the National Cancer Institute of Milan, Italy^7^. The most important risk factors associated with hepatic decompensation were, respectively, portal hypertension, extent of hepatic resection and MELD score greater than 9. The authors determined that patients without portal hypertension undergoing minor hepatectomy (resection up to two hepatic segments)^2^ are considered of low risk for decompensation of liver cirrhosis.

Another study evaluated data from the National Registry of the American College of Surgeons National Surgical Quality Improvement Program^31^. A total of 2,097 patients who underwent HCC resection were included in this study. Thrombocytopenia with a count below 100,000/mm^3^ was the most important adverse prognostic factor, conferring a mortality risk four times higher in multivariate analysis. Plaquetopenia with a count between 100,000 and 150,000/mm^3^ was also an adverse prognostic factor, with an 80% increase in the risk of postoperative mortality when compared with patients with normal platelet count. 

The quality of the remaining parenchyma is a key factor to be considered. The liver functional reserve (before surgery) and the regeneration capacity of the preserved parenchyma (after surgery) are major determinants for the risk of liver failure after partial resection^9^. The insufficient remnant liver function is defined as “small for size”, and is characterized by signs and symptoms of liver failure, prominent hyperbilirubinemia, encephalopathy and coagulopathy^9^.

**FIGURE 1:**
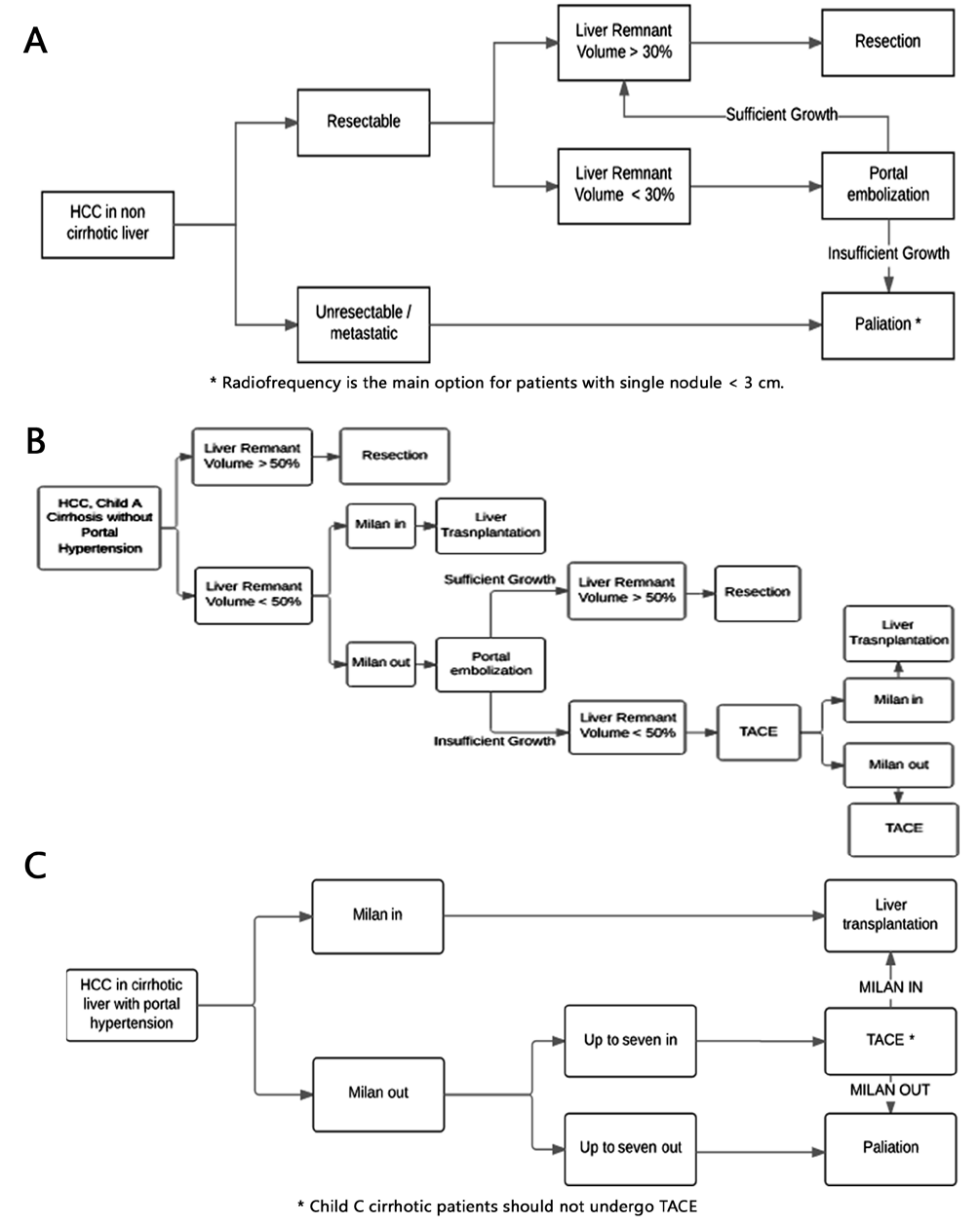
A) Suggested treatment algorithm for CHC patient without hepatic cirrhosis; B) suggested treatment algorithm for CHC in patients with Child A cirrhosis without portal hypertension; C) suggested treatment algorithm for CHC associated with cirrhosis with portal hypertension

In patients with normal remnant, the minimum amount of parenchyma that should be maintained after hepatectomy varies from 20-40% of the total original liver volume (Figure 1A). In addition to liver cirrhosis, other factors such as, advanced age, hepatic steatosis, presence of chronic viral hepatitis even without cirrhosis, previous chemotherapy with oxaliplatin or irinotecan and transoperative hemorrhage impair the regenerative potential of the liver remnant^9^.

At least 50% of the initial hepatic volume should be preserved in Child A patients with initial hepatic cirrhosis who are considered candidates for partial liver resections (Figure 1B)^9^. Patients with cirrhosis are generally not candidates for major partial hepatectomy. Child A patients without portal hypertension, especially those with small HCC, may be candidates for smaller partial hepatectomies. Whenever the location and size of the HCC allow, segmental or subsegmental resections are preferred (Table 4). The ideal surgical resection margin for HCC is 2 cm.

Due to the high risk of liver failure and death, Child C patients should not be submitted to partial hepatic resections. For these patients, therapeutic options with curative intent are restricted to LT, regarding that their HCC is within Milan criteria (Figure 1C).

**TABLE 4:** Resection criteria for HCC

No distant metastasis
Child A^*^
Absence of portal hypertension
Platelet count >100.000/mm^3^;
Liver remnant estimated by CT volumetry of the liver larger than 50% in cirrhotic patients
MELD ≤11
Karnofsky ≥ 70 (ECOG 0 or 1).

* Rarely, Child B patients are operated, provided they have small HCC and the other conditions above are fulfilled; ECOG=Eastern Cooperative Oncology Group

Management in cases in which remanescent liver is estimated as insufficient

When CT volumetry of ther liver suggests that the remnant is insufficient, patients without cirrhosis or with Child A stage cirrhosis and without portal hypertension may be submitted to embolization of the main branch of the portal vein on the same side of the tumor. Embolization is chosen to promote the growth of the hepatic remnant.

The quality of the remnant should also be assessed. In many cases, CT and/or MRI may be sufficient to assess the quality of the liver parenchyma not affected by the tumor. When major liver resections are planned, percutaneous biopsy of the hepatic lobe contralateral to the tumor may be utilized.

The procedure called ALPPS (Associating Liver Partition and Portal Vein Ligation) is another option in patients with large HCC and whose estimated liver remnant is small in size. In this procedure, the patient is submitted to laparotomy, ligation of the branch of the portal vein on the same side of the tumor and section (hepatotomy) of the area to be resected containing the tumor^1^. The abdomen is then closed, and the patient is reoperated one to four weeks later (depending on the evaluation of remnant growth), when the parenchyma portion containing the tumor is removed. ALPPS provides greater contralateral hypertrophy than isolated percutaneous radiological embolization, and is more commonly utilized for patients with liver metastases of colorectal cancer^10^.

### Adjuvant treatment following partial liver resection

In patients with viral hepatitis B and/or C, treatment of viral hepatitis following partial hepatectomy should be considered. New anti-viral drug regimens (eg, sofosbuvir, simeprivir, daclatasvir) may achieve more than 90% efficacy in the treatment of hepatitis C^29^.

Indication of the use of the tyrosine kinase inhibitor, sorafenib, in the treatment of HCC in advanced stages is well established^21^. However, its use as adjuvant therapy following partial post-hepatectomy for HCC is controversial. A randomized clinical trial comparing sorafenib to placebo involving 1114 patients submitted to adjuvant sorafenib therapy after partial hepatectomy or HCC ablation has been concluded^4^. There was no statistical difference in survival between the group of patients receiving sorafenib and the control group.

As the recurrence of HCC in the five years following surgical resection through partial hepatectomy is greater than 70%, chemotherapy after resection may be employed to address residual microscopic disease after resection. However, a meta-analysis evaluating a total of 108 patients enrolled in three randomized clinical trials of hepatic intra-arterial epirubicin followed by intravenous chemotherapy demonstrated chemotherapy to provide poor outcomes as adjuvant treatment for HCC^25^.

A recent meta-analysis evaluated the results of two randomized clinical trials and three case-control studies evaluating the effects of the use of I-131 lipiodol transarterial (via hepatic artery) for tumor chemoembolization (TACE)^15^. A total of 334 patients were included. This study detected a statistical benefit for patients receiving I-131 lipiodol over the control group^15^. However, this drug is not approved by the FDA and is not available for use in the US and most countries.

### Selection of patients for liver transplantation

Cirrhotic patients with HCC in the setting of advanced chronic liver disease are selectable for LT as long as not have distant metastases or nodal metastases are potential candidates for LT. Among those patients, those with single lesion up to 5 cm or up to three lesions of maximum 3 cm each (Milan Criteria) and absence of nodal or distant metastases may be listed at LT.^23,26,30^ Patients presenting with HCC larger than greater than 5 cm most tumors already show microscopic vascular invasion, which, in general, gives them a worse prognosis after LT^23^. Therefore, in Brazil and in many countries, listing patients whose HCC tumor disease is outside Milan criteria for LT is not allowed. Yet to be considered is the shortage of deceased donors, since in some states of Brazil, including the state of São Paulo, the waiting time for a liver may exceed one year^28^.

The majority of patients whose sum of the size of the largest HCC with the total number of tumors does not exceed seven (up-to-seven criteria) may undergo neoadjuvant treatment by TAE or TACE. Such treatment may be able to control and even decrease tumor mass in several patients (downstaging). A similar group of patients who may undergo TAE or TACE for the purpose of decreasing tumor disease are a large proportion of patients with HCC exceeding the Milan Criteria but still included in the UCSF Criteria (one tumor ≤6.5 cm, three tumors with the largest up to ≤4.5 cm, and a total tumor sum diameter ≤8 cm). If the tumor disease responds well to neoadjuvant treatment and is reduced to fit within the Milan Criteria (downstaging), these patients may be listed for LT.

In Brazil, since July 2006, the prioritization for LT of the patients listed for LT follows the criteria of MELD score severity. This score was developed at the Mayo Clinic, Rochester (MN), USA in 2002, constituting a logarithmic mathematical equation that estimates the risk of death over the next 90 days^19^. This equation uses serum INR, total bilirubin, and creatinine. MELD Score calculators are available on the internet.

MELD Score = 3.78 [Ln^*^ bilirubin (mg/dl)] + 11.2 [Ln^*^ INR] + 9.57 [Ln^*^ creatinine (mg/dl)] + 6.43 

* Ln=natural logarithm

Several patients with HCC under the Milan Criteria and listed for LT do not present an advanced degree of cirrhosis, presenting with only slightly elevated MELD scores. Although the degree of liver disease is not sufficient to give them an elevated calculated MELD score sometimes, neoplastic disease confers them a risk of death due to tumor spread. Thus, in Brazil and in several countries including the USA, patients listed for LT with HCC under Milan Criteria receive an assigned MELD score, denominated special criteria^17^. In Brazil, an appealed MELD score of 20 is attributed to patients with HCC larger than 2 cm at the time of listing. Three months after listing for LT, the assigned MELD score becomes equal to 24. After additional three months (six after listing), the appealed MELD score is 31, being maintained at that value until LT (or withdrawal of the patient from the waiting list by tumor progression or death).

### Management of tumor in patients waiting for liver transplant

Waiting time for LT is variable according to the country. Different centers employ different treatments to control HCC growth according to estimated wait time for the LT. Several invasive radiology procedures may be employed to control tumor disease. These include the transarterial chemoembolization TACE, TAE, radio frequency ablation (RFA) and percutaneous ethanol injection (PEI).

#### *Intra-arterial treatment: transarterial chemoembolization (TACE)/ transarterial embolization (TAE)*

Intra-arterial treatment promotes ischemic necrosis (coagulation necrosis) in the tumor. It is utilized for patients awaiting LT and also as palliation patients not selectable for resection or LT. It is contraindicated for Child C patients. It is indicated when there is more than one tumor nodule in a same hepatic lobe, for example. There are two forms of intra-arterial treatment: 1) transarterial embolization (TAE): the embolizing agent [polyvinyl acetate (PVA) or microspheres] is selectively injected into the tumor circulation through coaxial microcatheterism; 2) transarterial chemoembolization (TACE): lipiodol emulsified chemotherapy (usually doxorubicin, mitomycin C and cisplatin or combination) is selectively infused into the tumor circulation, followed by infusion of the embolizing agents (PVA or microspheres). Alternatively, the chemotherapy agent is infused simultaneously with specific drug-eluting beads, and interacts ionically with the chemotherapy agent.

Although TACE is generally preferred over TAE, there is no definitive evidence that TACE provides an increase in survival in relation to TAE. The response to intra-arterial treatment is monitored through abdominal CT scan with intravenous contrast. The treatment goal is to eliminate all neoplastic tissue inside the HCC nodule. Whenever there is detectable tumor tissue, TACE or TAE sessions are repeated at intervals of 60 to 120 days. TACE and TAE are good modalities of HCC control in patients listed for LT. Tumor progression is uncommon, occurring in less than 10% of cases, and allowing survival rates at five years post-transplantation reaching 70%^6^.

#### *Percutaneous ablation*

There are two techniques of percutaneous ablation: radiofrequency ablation (RFA) and chemical ablation with ethanol (PEI) or acetic acid. 

RFA allows thermal ablation of the tumor lesion, through an image-oriented needle is positioned close to HCC nodule and can also be performed through open surgery or laparoscopy. The tip of the needle is connected to a radiofrequency generator, and the radio waves are converted to heat inside the liver parenchyma. The tumor lesion is heated at elevated temperatures, promoting coagulation necrosis. RFA has been shown to be a safe option for these patients, and may provide similar results to those obtained with resection in BCLC stage 0 and A patients, with similar 3-year survival rates^3^. RFA can be employed for patients with HCCs measuring up to 5 cm as pre-transplantation therapy or even as palliative therapy. A contraindication to RFA is the presence major vessels or biliary branches in the vicinity of the tumor.

 Percutaneous ethanol ablation (PEI) uses ethanol (96° GL) that is infused through a needle inserted into the nodule through image guided percutaneous puncture. Ethanol promotes protein denaturation and cell death. PEI is a low-cost method that has demonstrated satisfactory results in HCC control of less than 3 cm and of superficial localization in the hepatic parenchyma^6^ ^,^ ^14^. PEI may be employed for Child A and B patients. It can also be utilized for palliation of unresectable HCCs in patients with Child A and B cirrhosis. Alternatively, acetic acid can be used for percutaneous treatment of HCC instead of ethanol. This therapy also shows results comparable to those obtained with PEI^32^. Because of its percutaneous application, both methods (injection of ethanol and injection of acetic acid) present a risk of tumor implantation in the needle track.

RFA promotes greater tumor necrosis and longer survival, with a significant reduction in local recurrence. However, recent meta-analyses only have detected survival benefit for RFA over PEI for HCCs smaller than 2 cm^14^.

### Results of hepatic resection vs. transplantation

LT for HCC may provide a 5-year survival reaching 70%^6^. Conversely, a recent study performed in Brazil revealed 5-year survival of 50% after partial liver resection for HCC^22^. The difference in survival rates between LT and resection for HCC may be due employment of different selection criteria between treatment methods. Unlike patients undergoing LT, as much as 50% of patients allocated to liver resection present with HCCs outside Milan Criteria^22^.

The option for either treatment takes into account the degree of impairment in liver function, HCC size, the stage of neoplastic disease, the estimated waiting time in the LT list and also the patient´s will. Post-transplant HCC recurrence risk is lower than recurrence risk following resection. Liver resection is generally preferred for patients with no cirrhosis or Child A cirrhosis, HCC size less than 3 cm, localization in anterior segments of the liver, and platelet count greater than 150,000 platelets/mm^3^. Recurrence can be treated with re-resections or with listing for LT (for patients who fulfill Milan criteria).

#### *Post-ressection or post-transplantation follow-up*

Post-resection HCC recurrence should be monitored, since there is a possibility of potentially curative treatment or even palliative treatment. There is no uniform protocol for follow-up of these patients. Different services utilize different protocols, but there is consensus that imaging studies either alone or associated with serum dosage of alpha-fetoprotein should be performed twice a year^7^. Abdominal CT and MRI are the most sensitive tests for detecting recurrence.

### Non-surgical treatment

Some procedures may be employed to control tumor progression in patients who do not meet the criteria for curative treatment through partial hepatectomy or LT. Some of these treatments (TAE, TACE, RFA, PEI and percutaneous acetic acid injection) also may be utilized as neoadjuvant treatments (see above).

RFA may offer disease control rates at 3 years similar to those of liver resection for patients whose HCC measures less than 3 cm^3^. Therefore, RFA is generally the preferred therapy for the treatment of HCCs in elderly and comorbidity patients who cannot be submitted to surgical treatment of HCC. In such cases, RFA can be utilized in combination with other modalities (TACE, TAE).

Other treatments are microwave therapy, transarterial radioembolization and cryotherapy. 

Microwave therapy is a method that, similar to RFA, promotes thermal tumor necrosis. Electromagnetic microwave therapy with a frequency greater than 900 kHz may achieve similar efficacy to RFA.

In transarterial radioembolization, yttrium-90 microspheres, I-131 labeled lipiodol or rhenium-1888 are injected into the tumor nodule via percutaneous transarterial route. Presence of portal vein thrombosis constitutes a contraindication to other forms of intra-arterial therapy. However, radioembolization is not contraindicated in patients suffering from portal vein thrombosis. 

In cryotherapy, multiple probes are inserted near the tumor nodule under US guidance. Ice at a temperature of -20º C promotes tumor cytotoxicity. Unlike radiofrequency ablation, cryotherapy may be employed in tumors located near to blood vessels.

HCC is a tumor that exhibits high expression of resistance genes that promote resistance to chemotherapy, including glycoprotein p and glutathione-S-transferase. HCC is therefore resistant to a vast majority of chemotherapy agents.

Molecular therapy through the tyrosine kinase inhibitor sorafenib has demonstrated a considerable (approximately three months) survival benefit for patients with advanced HCC over the placebo group^19^. In order to improve the performance of HCC palliative treatment, different treatment options can be utilized as combination.

## CONCLUSION

Hepatocellular carcinoma is potentially curable if discovered in its initial stages. Medical staff should be familiar with strategies for early diagnosis and treatment of hepatocellular carcinoma as a way to decrease mortality associated with this malignant neoplasm.

## References

[1] (2013). Associating liver partition and portal vein ligation for staged
hepatectomy (ALPPS) tips and tricks. J Gastrointest Surg.

[2] Bismuth H, Chiche L (1993). Surgery of hepatic tumors. Prog. Liver Dis.

[3] Bruix J, Reig M, Sherman M (2016). Evidence-Based Diagnosis, Staging, and Treatment of Patients With
Hepatocellular Carcinoma. Gastroenterology.

[4] Bruix J, Takayama T, Mazzaferro V (2015). Adjuvant sorafenib for hepatocellular carcinoma after resection
or ablation (STORM) a phase 3, randomised, double blind, placebo-controlled
trial. Lancet Oncol.

[5] Carrilho FJ, Kikuchi L, Branco F, Goncalves CS, Mattos AA, Brazilian HCC Study Group (2010). Clinical and epidemiological aspects of hepatocellular carcinoma
in Brazil. Clinics (Sao Paulo).

[6] Chedid MF, Scaffaro LA, Chedid AD (2016). Transarterial Embolization and Percutaneous Ethanol Injection as
an Effective Bridge Therapy before Liver Transplantation for Hepatitis
C-Related Hepatocellular Carcinoma. Gastroenterol Res Pract.

[7] Citterio D, Facciorusso A, Sposito C, Rota R, Bhoori S, Mazzaferro V (2016). Hierarchic Interaction of Factors Associated With Liver
Decompensation After Resection for Hepatocellular Carcinoma. JAMA Surg.

[8] Clavien PA, Lesurtel M, Bossuyt PM (2012). Recommendations for liver transplantation for hepatocellular
carcinoma an international consensus conference report. Lancet Oncol.

[9] Clavien PA, Oberkofler CE, Raptis DA, Lehmann K, Rickenbacher A, El-Badry AM (2010). What is critical for liver surgery and partial liver
transplantation size or quality?. Hepatology.

[10] Clavien PA, Petrowsky H, DeOliveira ML, Graf R (2007). Strategies for safer liver surgery and partial liver
transplantation. N Engl J Med.

[11] Colli A, Fraquelli M, Casazza G, Massironi S, Colucci A, Conte D, Duca P (2006). Accuracy of ultrasonography, spiral CT, magnetic resonance, and
alpha fetoprotein in diagnosing hepatocellular carcinoma a systematic
review. Am J Gastroenterol.

[12] Ebara M, Hatano R, Fukuda H, Yoshikawa M, Sugiura N, Saisho H (1998). Natural course of small hepatocellular carcinoma with underlying
cirrhosis. A study of 32 patients. Hepatogastroenterology.

[13] Forner A, Llovet JM, Bruix J (2012). Hepatocellular carcinoma. Lancet.

[14] Germani G, Pleguezuelo M, Gurusamy K, Meyer T, Isgrò G, Burroughs AK (2010). Clinical outcomes of radiofrequency ablation, percutaneous
alcohol and acetic acid injection for hepatocellular carcinoma: a
meta-analysis. J Hepatol.

[15] Hong Y, Wu LP, Ye F, Zhou YM (2015). Adjuvant Intrahepatic Injection Iodine-131 Lipiodol Improves
Prognosis of Patients with Hepatocellular Carcinoma After Resection a
Meta-Analysis. Indian J Surg.

[16] Hsu HC, Sep JC, Lin YH (1985). Prognostic histologic features of resected small hepatocellular
carcinoma (HCC) in Taiwan A comparison with resected large
HCC. Cancer.

[17] http://bvsms.saude.gov.br/bvs/saudelegis/gm/2009/prt2700_21_10_2009.html.

[18] Huguet C, Stipa F, Gavelli A, Blumgart LH (1996). Primary hepatocelular cancer: western experience. Surgery of the Liver and Biliary Tract.

[19] Kamath PS, Wiesner RH, Malinchoc M (2001). "A model to predict survival in patients with end-stage liver
disease". Hepatology.

[20] Kumar V, Abbas AK, Aster JC (2014). Robbins & Contran. Pathologic Basis of Diseases.

[21] Llovet JM, Ricci S, Mazzaferro V (2008). Sorafenib in advanced hepatocellular carcinoma. N Engl J Med.

[22] Lopes Fde L, Coelho FF, Kruger JA, Fonseca GM, Araujo RL, Jeismann VB, Herman P (2016). Influence of hepatocellular carcinoma etiology in the survival
after resection. Arq Bras Cir Dig.

[23] Mazzaferro V, Regalia E, Doci R (1996). Liver Transplantation for the treatment of small hepatocelular
carcinomas in patients with cirrhosis. N Engl J Med:.

[24] National Comprehensive Cancer Network (2016). NCCN Hepatobiliary Cancers Clinical Practice Guidelines in Oncology
Version 1.

[25] Ono T, Yamanoi A, Nazmy El Assal O, Kohno H, Nagasue N (2001). Adjuvant chemotherapy after resection of hepatocellular carcinoma
causes deterioration of long-term prognosis in cirrhotic patients:
metaanalysis of three randomized controlled trials. Cancer.

[26] SÁ Gustavo Pilotto D (2016). Liver transplantation for carcinoma hepatocellular in São Paulo:
414 cases by the milan/brazil criteria. ABCD, arq. bras. cir. dig.

[27] Salem R, Lewandowski RJ (2013). Chemoembolization and radioembolization for hepatocellular
carcinoma. Clin Gastroenterol Hepatol.

[28] Salvalaggio P, Afonso RC, Pereira LA, Ferraz-Neto BH (2012). The MELD system and liver transplant waiting-list mortality in
developing countries lessons learned from São Paulo, Brazil. Einstein (Sao Paulo).

[29] Terrault NA, Zeuzem S, Di Bisceglie AM (2016). Effectiveness of Ledipasvir Sofosbuvir Combination in Patients
With Hepatitis C Virus Infection and Factors Associated of Sustained
Virologic Response. Gastroenterology.

[30] Torres OJ, Marques MC, Santos FN, Farias IC, Coutinho AK, Oliveira CV, Kalil AN, Mello CA, Kruger JA, Fernandes GD, Quireze C, Murad AM, Silva MJ, Zurstrassen CE, Freitas HC, Cruz MR, Weschenfelder R, Linhares MM, Castro LD, Vollmer C, Dixon E, Ribeiro HS, Coimbra FJ (2016). Brazilian consensus for multimodal treatment of colorectal liver
metastases. Module 3: controversies and unresectable
metastases. Arq Bras Cir Dig.

[31] Venkat R, Hannallah JR, Krouse RS, Maegawa FB (2016). Preoperative thrombocytopenia and outcomes of hepatectomy for
hepatocellular carcinoma. J Surg Res.

[32] Weis S, Franke A, Berg T, Mössner J, Fleig WE, Schoppmeyer K (2015). Percutaneous ethanol injection or percutaneous acetic acid
injection for early hepatocellular carcinoma. Cochrane Database Syst Rev.

